# Systematic review of the impact of feed protein type and degree of hydrolysis on gastric emptying in children

**DOI:** 10.1186/s12876-015-0369-0

**Published:** 2015-10-15

**Authors:** Rosan Meyer, Ru-Xin Melanie Foong, Nikhil Thapar, Stamatiki Kritas, Neil Shah

**Affiliations:** 1 Gastroenterology Department, Great Ormond Street Hospital for Children NHS foundation Trust, London, UK; 2 Institute of Child Health, University College, London, UK; 3 KU Leuven, TARGID, Leuven, Belgium

**Keywords:** Gastric emptying, Feed, Formula, Hydrolysed and whole protein

## Abstract

**Background:**

The choice of infant formula is thought to play an important role on gastric emptying (GE) in a variety of gastrointestinal disorders. It is known that many ingredients impact on GE, including the type of protein and level of hydrolysis. In clinical practice, feeds are often recommended due to putative improved GE related to the type of protein and level of hydrolysis, however whether this is scientifically justified still needs to be established. A systematic review comparing the impact of protein type and hydrolysis on GE in children was therefore performed.

**Methods:**

The Patient, Intervention, Comparison and Outcome system was used. A structured literature search was performed using the Preferred Reporting Items for Systematic Reviews and Meta-analysis guidelines, searching PubMed, Cochrane databases and Google Scholar from 1990 to 2014. We only included articles published in full text English language using specific search terms, including both scintigraphy and C13-octanoic acid breath test.

**Results:**

We identified 126 publications of which 20 were eligible for inclusion but only 8 were included. Studies reviewed GE in both healthy children as well as those with neurodevelopmental delay and reflux. Two studies investigating GE of breast milk versus formula indicated a faster GE for breast milk. Four studies found that feeds containing whole whey in varying amounts emptied faster than predominant whole casein feeds and one study found no difference in GE. Five studies investigated a mix of whole versus hydrolysed protein and found conflicting results related to study population and hydrolysis.

**Conclusions:**

Breast milk has a faster GE than formula milk. Although there seems to be a trend towards whey feeds emptying faster, different methodologies, feed compositions and patient groups makes it difficult to draw firm conclusions. Future studies should be performed with comparable feeds in populations where increased GE may be of clinical benefit.

## Background

Breast milk remains the gold standard source of nutrition for infants due to both its unique nutritive and non-nutritive ingredients [1]. For infants where breast milk is not available, infant formulas with a variety of characteristics are now widely accessible for both healthy infants and those with underlying conditions affecting the gastrointestinal tract or metabolic function [2]. The impact of infant formulas on gastric emptying (GE) plays an import role in the choice of feed for children with gastro-oesophageal reflux (GOR), gastroparesis and also dysmotility disorders [3, 4]. It is known that a variety of factors impact on GE in paediatrics, including the type of protein (i.e. whey or casein), level of hydrolysis, the amount and type of fat, energy density, viscosity, fibre content and osmolality [5–9]. For liquids it is thought that the energy content is the primary determinant of GE [10]; however a study by Khoshoo et al. [8] showed similar GE of a high and low energy whey based feed, suggesting that protein source may be of even greater importance. In other studies the level of protein hydrolysis has also been shown to accelerate GE and improve gastrointestinal symptoms in children with reflux [4, 11].

Studies have implemented a variety of methods to assess GE in various patient populations, including sonography, MRI, electrical impedance, double sampling aspiration technique, paracetamol absorption, scintigraphy as well as breath test (BT) [9, 12–14]. However, not all techniques have yielded reproducible data when tested in paediatrics: sonography is operator dependent and the observation time is shorter, paracetamol absorption technique is not used in infants and electrical impedance, although a good correlation with scintigraphic methods produces considerable noise [14–16]. Scintigraphy has long been considered the gold standard for assessing GE and has been validated in the paediatric setting [13]. It is a minimally invasive, low cost physiological methodology in which a radiolabelled liquid or solid meal (with Tc99 nanocolloid or sulphur colloid or ^99m^Tc-Diethylenetriaminepentacetate [DTPA]) is imaged and quantified. Although widely in use, like many paediatric tests, there is a lack of normative values across the age groups and some have expressed concerns in regard to radiation exposure. More recently, BT have been used as a non-radioactive alternative to scintigraphy, measuring stable isotope in serial expired breath following ingestion of an isotope labelled meal [17]. The ^13^C-Octanoic Acid breath test (13C-OABT) has been used extensively to study GE of a variety of feeds and has shown to correlate well with GE half-emptying time (t1/2) established with scintigraphy [18, 19].

Given breast milk predominantly contains whey protein, studies have focused on whey-based formulas, which are thought to be more easily digested and promote faster GE compared to casein-based protein formulas [11]. However, infant feeds are a composite of protein, energy, carbohydrates and fats that all impact on GE. A previous review of these studies have also revealed significant variation in experimental designs, feeds used and have yielded conflicting results [9]. Consequently the GE benefit based on the type of protein and the impact of hydrolysis remains unclear. We therefore aimed to compare GE of breast milk to predominant whole casein and whey formulas and the latter to hydrolysed whey or casein formulas.

## Methods

### Identification and retrieval of literature

The Patient, Intervention, Comparison and Outcome (PICO) system was used for the outline of this systematic review. A structured literature search was performed using the methods and procedures of the Preferred Reporting Items for Systematic Reviews and Meta-analysis (PRISMA) guidelines. We used PubMed, Cochrane databases and Google Scholar from January 1990 to December 2014 using the following specific search terms: gastric emptying [tiab/tw] AND formula/feed [tiab/tw] AND children/paediatric [tiab/tw] OR gastric emptying [tiab/tw] AND protein [tiab/tw] AND children/paediatric [tiab/tw] OR gastric emptying [tiab/tw] AND whey [tiab/tw] OR casein [tiab/tw]. We excluded unpublished work, as well as conference abstracts but included published full text English language peer reviewed studies with the following study design (Table 1):

**Table 1:** Systematic review inclusion and exclusion criteria for GE studies using formula/enteral feeds or breast milk

Inclusion criteria	Exclusion criteria
Patient cohort	Patient cohort
All studies on preterm infants	Adult studies (>16 years)
All studies on children (birth – 16 years)	Animal studies
Intervention	Intervention
Whole protein formula or feeds	Paediatric formula/feeds assessing impact of osmolality, carbohydrates and fat content on GE
Partial or extensively hydrolysed casein formula or feeds	
Partial or extensively hydrolysed casein formula or feeds	Paediatric formula/feeds that are mixed at a non-standard concentration
Mix of casein and whey protein, hydrolysed or whole formula or feeds	Paediatric formula/feeds that are pre-thickened
	Adult feeds
Amino acids formula or feeds	Any feeds tested in animal models
Comparison	Comparison
Whole protein formulas versus breast milk	Adult feeds
Whole protein versus hydrolysed (casein/whey) formula/feeds	Animal models
Hydrolysed (casein/whey) versus amino acid formula/feeds	
Amino acid versus whole protein formula/feeds	
Outcome measurements	Outcome measurements
Scintigraphy (Tc99 sulfur colloid scan)	Paracetamol absorption
13C-OABT	13C-acetate BT
13C-Na- OABT	Ultrasound
	Manometry

1. Randomized controlled trials (RCTs).
2. Non-randomized controlled clinical trials (NRCT).
3. Before and after clinical trials (CT).
4. Observational studies i.e. cohort (CS) or case reports (CRs).

We have included studies that used sulfur colloid (Tc99) scintigraphy and studies using stable isotope BT (13C-OABT and 13C-Na OABT) as paediatric studies have shown both as reliable method [20, 21]. Publications were included if they were performed in both preterm and paediatric populations (up to 16 years of age) and compared GE of breast milk or other formulas with whole or hydrolysed casein or whey. Due to the limited publications available, studies both in healthy and sick children were included. Further details on inclusion and exclusion criteria are in Table 1.

The quality of the studies were assessed by RF and SK, and verified by RM who was the third assessor. Studies were assessed using the SIGN (Scottish Intercollegiate Guidelines Network) criteria. When there was disagreement on the inclusion/exclusion of a publication this was discussed with NS, the supervisor of the study, who was not involved in the search of publications.

We aimed to assess GE of:

1. Breast milk versus whole casein/whey formulas
2. Whole casein versus whole whey formulas
3. Whole protein versus hydrolysed protein
4. Hydrolysed casein versus hydrolysed whey

## Results

**Fig. 1:**
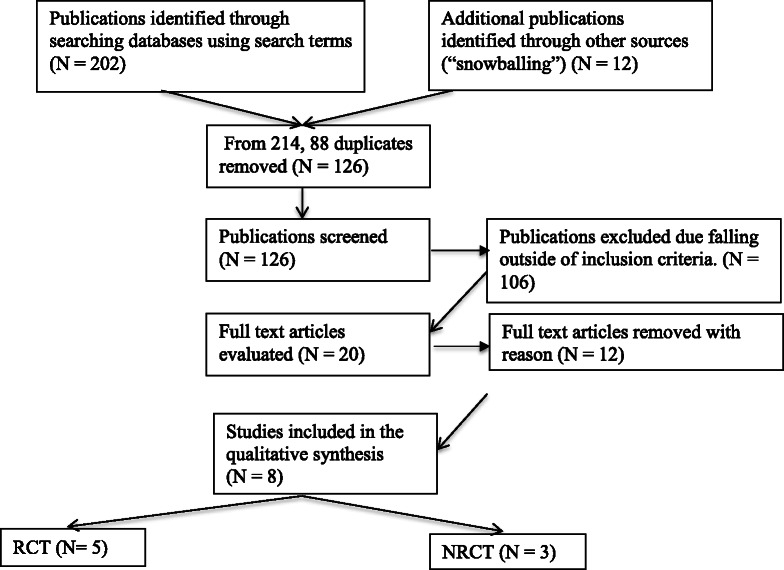
Study selection for the systematic review. *RCT randomised controlled trial NRCT non randomised controlled trial

We identified 202 articles with the literature search and 12 further studies through “snowballing” (additional articles identified through other sources). From the total of 214 publications, we removed 88 duplicate publications and RF and SK screened 126 publications. Twenty suitable studies were within our inclusion criteria and were discussed with RM: 10 were excluded due to the study not being aimed at establishing differences in GE of feeds related to protein content, or using only breast milk, comparing different hydrolysed feeds only or insufficient information on feeds used. Of the remaining publications, 4 were further discussed with NS (external gastroenterologist), with 2 of these subsequently excluded, resulting in only 8 studies deemed suitable for review. Finally only 8 studies were deemed suitable for the review using either scintigraphy or BT and with sufficient feed information to answer our research questions (Fig. 1). There were five studies that measured GE using the 13C-OABT, and three studies that used Tc-99 sulfur colloid scintigraphy. We identified no CR and CT, but 6 RCT (4 cross-over studies) and 2 NRCT. Two of the studies (1 NRCT and 1 RCT) included preterm infants (chronological age < 37 weeks) and the rest were conducted in children (Fig. 1). Of the included studies 3 were performed in children with cerebral palsy (CP), 2 in children with gastroesophageal reflux and 3 in healthy infants/preterm infants.

### Breast milk versus whole whey/casein feeds

Only 2 publications were found that used either scintigraphy or 13C-OABT to compare GE of breast milk to whole formula milks. Van Den Driessche et al. [20] compared breast milk and formula using scintigraphy in 29 preterm infants. A matched volume of 50 ml whole protein infant formula containing a ratio of 60 % whey to 40 % casein was compared to expressed breast milk. The study concluded that GE was significantly faster in breast-fed compared to formula-fed infants (*p* < 0.05; t1/2 47 min vs 65 min). However, there was a 3-week difference in the gestational age between the two groups, with the mean gestational age of the breast-fed infants being 36 weeks compared to 33 weeks in the formula-fed group, which potentially could have influenced the results (Table 2). In addition this study did not measure the protein composition and whey:casein ratio of breast milk, but assumed its content which may have affected study results.

**Table 2:** Summary of GE studies included in this systematic review

Author and year	Study design and sample	Method	Test feeds	Results
Van den Driessche et al. [20] 1999	NRCT (*n* = 29)	13C-OABT	Test feed 1: Breast milk	Test feed 1: t1/2 47 min
	Preterm infants (27–41 weeks)		Test feed 2: 40 % casein, 60 % whey (Nutrilon Premium, Nutricia)	Test feed 2: 65 min (*p* < 0.05)
Savage et al. [22] 2012	RCT cross-over (*n* = 13) on children with CP	13C-OABT	Test feed 1: 82 % casein-based 82, 18 % whey (Paediasure, Abbott)	Test feed 2 and 3: t1/2 33.9 min
			Test feed 2: 50 % casein, 50 % whey [Nutren Junior, Nestle Clinical Nutrition]	Test feed 1: 56.6 min (*p* = .033)
	Age: 2.4–15.4 years			
			Test feed 3: 100 % pHF^a^ whey [Peptament Junior, Nestle Clinical Nutrition]	
Brun et al. [7] 2012	RCT cross-over (*n* = 15) children with CP	13C-OABT	Test feed 1: 100 % casein	Test feed 1: t1/2 153 min, Test feed 2: t1/2 82
			Test feed 2: 100 % hydrolysed whey	
			Test feed 3: 100 % amino acids	Test feed 3: 74.4 min
	Age: 6–16 years		Test feed 4: 40 % casein, 60 % whey	Test feed 4: 63.3 min Fastest GE was for test feed 4 (*p* < 0.001)
			Test feeds contained standard carbohydrate and fat	
Brun et al. [30] 2013	RCT (*n* = 10) children with CP with a Nissen fundoplication versus (*n* = 10) children with CP but without Nissen fundoplica-tion	13C-OABT	Test feed 1: 100 % casein	t1/2 test feed 1:
			Test feed 2: 40 % casein, 60 % whey	- 110 min for Nissan fundoplication group
				- 181 min for non Nissan fundoplication group
				t1/2 test feed 2:
				- 50 min for Nissan fundoplication group
				- 85 min for non Nissan fundoplication group
				In both groups feed 2 emptied faster
Staelens et al. [24] 2008	RCT cross-over (*n* = 20) healthy infants	13C-OABT	Test feed 1: 29 % casein, 71 % whey (NAN 1, Nestle)	Test feed 3: t1/2 46 min Test feed 1: t1/2 55 min
			Test feed 2: 100 % pHF^a^ whey (NAN HA, Nestle)	(*p* =0.036)
	Age: 6–13 weeks			No difference between t1/2 between test feed 1 and feed 2
			Test feed 3: 100 % eHF^b^ whey formula (experimental Nestle formula)	
Thorkelsson et al. [23] 1994	RCT (*n* = 20) preterm infants Age: 33–34 weeks	Tc-99 scintigraphy	Test feed 1: 40 % casein, 60 % whey (Similac Special Care, Ross Laboratories)	Test feed 1: t1/2 64.9 ± 12.3 min
				Test feed 2: t1/2 56.5 ± 14.8 min
			Test feed 2: 82 % casein,18 % whey (experimental formula, Ross Laboratories)	No significant difference in GE between formulas. (*p* = 0.75)
Tolia et al. [25] 1992	RCT cross-over (*n* = 28) infants with reflux Age: infants < 1 year of age	Tc-99 scintigraphy	Test feed 1: 82 % casein:18 % whey (Similac, Ross Laboratories)	Test feed 1: t1/2 39.7 ± 2.02 min
			Test feed 2: whole soya formula (Isomil, Ross Laboratories)	Test feed 2: t 1/2 44.6 ± 2.01 min
			Test feed 3: 100 % whey hydrolysate (Goodstart, Carnation Company)	Test feed 3: t1/2 48.5 ± 2.89 min.
				GE of feed 3 was significantly (*p* < 0.05) slower 48.5 % versus 39.7 %
Billeaud et al. [11] 1990	NRCT (*n* = 111) infants with GOR and (*n* = 90) healthy controls	Tc99 scintigraphy	Test feed 1: breast milk	At 30 min no difference in GE between formulas.
			Test feed 2: 100 % hydrolysed whey (Nidal HA, Nestle)	
				Gastric residual content at 120 min was 18 +/− 11 % with breast milk, 16 +/− 21 % feed 2, 25 +/− 17 % feed 3, 26 +/− 19 % feed 4, 39 +/− 17 % feed 5, 47 +/− 19 % feed 5, 55 +/− 19 % feed 7
			Test feed 3: acidified whole protein (Pelargon, Nestle)	
	Age: <1 year of age (range not specified)		Test feed 4: 40 % casein, 60 % whey (Lactamil, Jaquemaire)	
			Test feed 5: 80 % casein, 20 % whey (Alma, Jaquemaire)	
			Test feed 6: 80 % casein, 20 % whey Follow-up formula (Nido, Nestle)	
			Test formula 7: whole cow’s milk (80 % casein)	

^a^pHF = partially hydrolysed formula, ^b^ eHF = extensively hydrolysed formula

**Table 3:** Key summary points

1.	Breast milk empties the stomach faster than whole protein infant formula.
2.	Predominant whole casein feeds empty slower when compared to predominant whey feeds in children with CP and GOR.
3.	Differences in GE data exists between healthy children and those with underlying conditions.
4.	Whole versus hydrolysed protein may affect children differently depending on their underlying diagnosis and age.
5.	No data exists on the GE of extensively hydrolysed casein versus partially hydrolysed casein formulas.
6.	Studies utilise a variety of different feeds, with varying compositions in different populations, it is therefore not possible the draw firm conclusions on GE for all children in regard to feed protein type and hydrolysis.

Billeaud et al. [11] compared GE using Tc99 scintigraphy of 110 infants with versus 90 without GOR. The infants were fed breast milk or a variety of standard formulas and cow’s milk (Table 2). In the pooled data from both groups, gastric residual content at 120 min was 19 ± 16 % for breast milk, 25 ± 18 % for whey-predominant formula (60 % whey) and 38 ± 21 % for casein-predominant formulae (80 % whey) and 46 ± 19 % with the follow-up formula, which is also casein dominant. Breast milk emptied the fastest for both infants with and without reflux in comparison to predominant casein and whey feeds. Possible bias may have been introduced by not controlling for the volume consumed prior to the scintigraphy (ranging from 110 ml 1 month- 200 ml at 1 year) and the osmolality of the casein dominant feed was significantly higher (350–380 mOsm/kg H2O) than the whey dominant feed (290–300 mOsm/kg H2O). Additionally the protein content of the follow-on formula was significantly higher than other formulas and similar to the study by Van den Driessche et al. breast milk protein content was not measured.

### Whole protein casein versus whey feeds

We identified 5 published studies that compared a varying mixture of whole casein to whey formulas. Savage et al. [22] in 2012 conducted a pilot study in 13 children with CP who were enterally fed. Each child served as their own control with random crossing over between feeds. The volume provided to each patient was the same and the protein content was well matched, but there were slight differences in carbohydrate and fat between these feeds. Patients received a predominant casein-based enteral formula [Pediasure, Abbott (82 % casein, 18 % whey)] for 1 week, followed on by a feed containing a 50 % mix of whole-whey and casein protein [Nutren Junior, Nestle Clinical Nutrition] (*n* = 7) or a whey hydrolysate (results not reported in this section). The authors of this study found that the feed containing 50 % whole whey had the fastest GE (t1/2 33.1 min) when compared to the casein predominant feed (t½ 56.6 min). Although the whole whey formula emptied faster, statistical significance was not established between these feeds due to the small number of patients, only when GE data for hydrolysed whey feed and the 50 % whole whey feed was combined was statistical significance achieved.

Brun et al. [7, 17] published two studies in children with CP (one in children with and without Nissans Fundoplication) that compared 100 % whole casein to a predominant whole whey protein feed (40 % whey and 60 % casein). Feeds used in both studies were 100 % matched for volume (200 ml), energy (1 kcal/ml), protein (2.8 g/100 ml), carbohydrates (12 g/100 ml) and fats (4.5 g/100 ml) and used the same nutrient sources except for the type of protein. In both studies the predominant whole whey feed emptied significantly faster than the 100 % casein feed. The studies by Brun et al. [7, 17] have the advantage over other studies, that all feeds were 100 % matched ingredients except for the protein source which differed, which significantly reduces bias.

Billeaud et al. [11] compared the GE of a variety of standard formulas in children with/without GOR, including a predominant whole whey (60 % whey) and casein formula (80 % casein) and a follow-on formula that was predominant casein as well. GE did not differ with age or gender, but differed mainly according to the type of feed. At 30 min, there was no difference between the predominant casein or whey formulas in the pooled data. However, at 120 min the predominant whey formula had a significantly (*p* < 0.05) faster GE (25 ± 18 %) than the predominant whole casein formulas, at 38 ± 21 % and 46 ± 19 % for the follow-on formula.

The study by Thorkelsson et al. [23] compared GE in 20 healthy preterm infants on either a predominant whey or casein formula. This is the only study reported in this review that found no significant difference in GE between the two formulas with t1/2 being 64.9 ± 12.3 min for the whey dominant formula and 56.5 ± 14.8 min for the casein dominant formula. Although this study does not provide the macronutrient content of feeds used, the authors mention that feeds were the same except for protein composition.

### Whole protein compared to hydrolysed protein (casein versus whey) feeds

To date no studies have been published assessing the GE of hydrolysed casein versus whole protein feeds in children using validated methods. Five studies compared GE of whole protein to hydrolysed whey protein in feeds, of which two publications documented also their comparison of whole protein feeds (varying casein and whey) to either amino acid or soya feeds, which will be mentioned in this review but not discussed. The study conducted by Savage et al. [22] compared GE of a predominant whole casein formula to a 50 % whey and casein mixture and a partially hydrolysed (pHF) whey feed in children with CP. In that study the formula containing 50 % whole whey had a faster GE (t1/2 33.1 min) than the 100 % pHF whey feed (t1/2 39 min). However due to the small number of patients in the study, there was no statistical significance between whole protein feeds and the pFH whey. Statistical difference was only achieved when whey formulas (pHF whey and whole whey) were grouped together (GE t1/2 34 min vs 57 min; *p* = 0.033). In addition there were marked differences in osmolality and percentage of medium chain triglycerides between these two feeds.

Staelens et al. [24] 2008 compared three formulas in healthy infants of varied hydrolysis and protein type but with similar nutritional content for energy, protein, fat and osmolality. There was a slight difference in carbohydrate content, in particular related to the amount of lactose, which may have affected study results. A predominant whole whey protein feed was compared to a 100 % pHF whey and a 100 % extensively hydrolysed (eHF) whey feed. The results showed significantly faster GE of eHF whey (t1/2 46 min) compared to both the pHF whey and whole protein feed (t1/2 55 min) in healthy children (*p* = 0.019 and *p* 0.008 respectively), but there was no difference in GE between the whole protein feed and pHF whey (t1/2 53 min).

In the study by Billeaud et al. [11], a 100 % hydrolysed whey formula was compared to a whole predominant casein or whey formula in children with and without GOR. The GE for the hydrolysed formula was 21 ± 19 % for the pooled population and significantly faster (*p* = 0.05) than both whole casein and whey formulas. The limitation of this study was that the level of hydrolysis was not stated for the whey hydrolysate, which may have affected the outcome. In addition volume of feed consumption differed per age and protein, osmolality, fat and lactose content differed that may have affected the results.

The study by Tholia et al. [25] aimed to assess GE in infants with established GOR. Infants acted as their own controls as they were randomly crossed over to different feeds, which included a whole predominant casein feed, whey hydrolysate and soya formula. Unlike the aforementioned studies, the GE t1/2 was significantly faster in the whole casein feed (39.7 ± 2.02 min) when compared to the whey hydrolysate (48.5 ± 2.8 min). This study also does not indicate the level of hydrolysis and the authors were not able to establish this from company information due to the study being completed in 1992. Nutrient content of feeds varied significantly, including osmolality, fat composition and ratio of medium chain triglycerides.

In the study by Brun et al. [7] on children with CP using C13-OABT and carbohydrate/fat matched feeds, the authors found that the whole casein formula (t1/2 153 min) had the slowest GE compared to a hydrolysed whey formula (t1/2 82 min), amino acid formula (t1/2 74 min) and whey:casein mixture (t1/2 63.3 min). The hydrolysed whey emptied significantly faster (*p* 0.08) than the whole casein feed, however the whole casein:whey mixed feed emptied faster than the hydrolysed whey formula (*p* value not stated). Although feeds were matched in macronutrients, the authors did not state the level of hydrolysis (pHF or eHF) or osmolality differences, which occur when hydrolysed protein is used.

## Discussion

To the best knowledge of the authors this is the first systematic review aiming to assess the impact on GE in paediatrics of both the type of protein and level of hydrolysis in paediatric feeds and considers GE of breast milk as well. Our findings with regard to protein type concur with those of Woodley et al. in 2008, but add further information on GE and level of hydrolysis [9]. This review identified 8 paediatric studies using current validated methods of scintigraphy or 13C-OABT in both healthy children, those with neuro-disabilities and reflux.

We identified 2 studies that found increased GE with breast milk versus whole protein formula milk. The study by Van Den Driessche et al. [20], however, did have significant differences in age between the two studied groups, which could have affected the result. Billeaud et al. [11] compared the GE of breast milk to five other feeds, including a predominant whole whey formula and two predominant casein feeds. In all aforementioned feeds, GE was slower in the whole protein feeds than in breast milk. Although only 2 studies with a total of 230 infants on either breast milk or a variety of infant feeds were suitable for inclusion, the results seem to indicate that breast milk empties faster than whole milk formulas. The superior emptying properties of breast milk, seemed to be maintained even with increasing volumes, according to a study by Pozler et al. [26]. Conceptually this superior GE pattern with breast milk can be explained due to its high whey content with faster GE properties, unique fat blend, lower osmolality than whole formula milk, and most importantly non-nutritive factors, including amylase, that may aid digestion and gastric emptying [27–29].

In nutritional practice it is often recommended to use whey protein for better GE in children with disorders that affect motility of the gastrointestinal tract. The purported benefits of whey protein relate to the predominance of β-lactoglobulin, which remains soluble in the stomach, therefore transiting more rapidly to the upper jejunum [29]. In the past there have been concerns also that casein dominant feeds cause lactobezoars due to slower gastric emptying in preterm infants [23]. In our systematic review we found 5 studies, that each included between 10–20 children, investigating GE of predominant whole casein versus whey protein feeds [7, 11, 22, 23, 30]. In none of these studies 100 % casein feeds were compared to 100 % whey feeds, but instead comparisons of predominant whole casein feeds to whole protein feeds containing either 50 % or 60 % whole whey. In 4 studies, the predominant whole whey feeds emptied faster than the predominant casein feeds, however Thorkelsson et al. [23] found no difference in GE between predominant whole casein or whey feeds. The opposing outcome of the studies could be explained by differences in feed composition but also by the difference in study population. Those studies that found a difference in GE between type of protein occurred in children that either had GOR or CP, with GOR also being prevalent in the latter cohort [31]. Predominant whole whey protein therefore may have a faster GE in children that have underlying GOR, but not healthy children. Further studies are required to answer this question and should implement the method from Brun et al. [7] matching volume, carbohydrates, fats, energy and only varying protein content. To date no paediatric study has compared GE of 100 % whole whey and 100 % whole casein feeds, likely due to the unavailability of commercially prepared 100 % casein based formula. Currently only mixes of whey and casein have been used suggesting that a predominant whey feed (that also contains casein) may have better GE properties in GOR (Table 3).

On the question of the impact on hydrolysis 5 studies looked at the effect on GE. Although the study by Savage et al. [22] did find different GE pattern between pHF whey and whole protein feeds, it was only the combined data of whole predominant whey and pHF whey that yielded a statistically significant result and did therefore not add further data to the question at hand. Staelens et al. [24] found that a 100 % eHF whey emptied faster than both pHF whey and whole protein feed in healthy infants. Billeaud et al. [10] also found that in infants with/without GOR the hydrolysed whey emptied faster at 120 min than all other whole protein formulas. Brun et al. [7] on the other hand found that hydrolysed whey protein emptied slower than a mix of 60:40 whole whey and casein feed and also an amino acid formula in children with CP. Similarly, Tolia et al. [25] also found that in children with GOR, the whey hydrolysate had a significantly slower GE when compared to the whole whey casein mixed feed. The findings seem to contradict each other and may be explained by the different study populations (healthy versus CP/GOR), the age (infants versus older children) and also by feeds that were not optimally matched. Most importantly the level of hydrolysis was not specified in 3 of the studies and Staelens et al. [24] clearly showed a difference between pHF and eHF whey in GE. In addition there are significant differences in feed composition in some of the studies. It is therefore difficult to draw any firm conclusion on the question of GE between whole and hydrolysed protein (Table 3).

The limitations of this review include the very small number of publications that have been published using either scintigraphy or 13C-OABT as methods for GE. Although these methods have been validated, it is important to be aware of their limitations. The most pervasive pitfall in GE by scintigraphy is the use of short-duration detailed studies, lasting 2 h, and extrapolating the t1/2 or the proportion emptied at 4 h using a power exponential analysis. For the 13C-OABT, pitfalls include potential loss of accuracy in patients with other diseases involving the intestinal mucosa, pancreas, liver, and respiratory system. On the other hand C13 octanoic acid is absorbed across the mucosa undigested, so it is not dependent on biliary and pancreatic secretions or mucosal enzymes [32]. Other methods for gastric empting in children have been used such as imaging tests (i.e. magnetic resonance imaging, functional ultrasonography) and imaging procedures, which have had good results; however, the ability to ensure consistency between the studies using this method is less reliable and often require skilled technicians [13, 32, 33]. Therefore, although other proxy methods for gastric emptying in children exist, it would have made any comparisons difficult due to the difference in measurement techniques and varying reliability. Further limitations of this systematic review which reduces its ability to draw firm conclusions, are the small sample sizes in the majority of studies, the varying feed composition (i.e. varying fat and osmolality), level of hydrolysis, volume consumption and different patient cohorts (healthy versus unwell) that these feeds have been tested in including preterm infants that have different GE to full term infants [6, 34–36]. The majority of the aforementioned limitations have also been highlighted by a previous review by Woodley et al. [9] and have not been optimally addressed by subsequent studies.

## Conclusion

This systematic review aimed to assess the impact of the type and the hydrolysis of protein on GE. Only a small number of studies have been identified using current gold standard techniques and therefore limited conclusions can be drawn from this number of studies. It does however seem that breast milk has a faster GE in comparison with whole protein formula milk and feeds that contain whole whey appear to empty faster, however this may be affected by the underlying diagnosis. It is difficult to provide guidance on the impact of hydrolysis on GE due to the varying study designs and feed ingredients. Most importantly, this review has highlighted the paucity of feed data in the area of protein and level of hydrolysis on GE. We have also pointed out the difficulties in an optimal study design in regard to GE that compares feeds of similar energy density, fat, protein content and osmolality. Future studies should aim to compare feeds with the same energy density, type of fat, and comparable levels of casein/whey and extent of hydrolysis in target populations where faster GE may assist in symptom relief.

## Abbreviations

BT: Breath test
CP: Cerebral palsy
CR: Case reports
CS: Cohort study
CT: Clinical trials
eHF: extensively hydrolysed formula
GE: Gastric emptying
GOR: Gastro-oesophageal Reflux
NRCT: Non-randomized controlled clinical trials
pHF: partially hydrolysed formula
RCT: Randomised controlled trial
t1/2: gastric half-emptying time
